# Challenges in using ^18^ F-fluorodeoxyglucose-PET-CT to define a biological radiotherapy boost volume in locally advanced pancreatic cancer

**DOI:** 10.1186/1748-717X-9-146

**Published:** 2014-06-24

**Authors:** James M Wilson, Somnath Mukherjee, Kwun-Ye Chu, Thomas B Brunner, Mike Partridge, Maria Hawkins

**Affiliations:** 1 CRUK/MRC Oxford Institute for Radiation Oncology, Gray Laboratories, University of Oxford, Old Road Campus Research Building, Off Roosevelt Drive, Oxford OX3 7DQ, UK; 2 Department of Radiation Oncology, University Hospitals of Freiburg, Robert-Koch-Str. 3, D-79106 Freiburg im Breisgau, Germany

**Keywords:** Pancreatic cancer, PET-CT, Residual metabolic activity, Intra-tumour heterogeneity, Biological target volume

## Abstract

**Background:**

The best method of identifying regions within pancreatic tumours that might benefit from an increased radiotherapy dose is not known. We investigated the utility of pre-treatment FDG-PET in predicting the spatial distribution of residual metabolic activity following chemoradiotherapy (CRT) in locally advanced pancreatic cancer (LAPC).

**Methods:**

17 patients had FDG-PET/CT scans at baseline and six weeks post-CRT. Tumour segmentation was performed at 40% and 50% of SUV_max_ at baseline and 60%, 70%, 80% and 90% post-CRT. FDG-PET scans were non-rigidly registered to the radiotherapy planning CT using the CT component of the FDG-PET/CT. Percentage overlap of the post-CRT volumes with the pre-CRT volumes with one another and the gross tumour volume (GTV) was calculated.

**Results:**

SUV_max_ decreased during CRT (median pre- 8.0 and post- 3.6, p < 0.0001). For spatial correlation analysis, 9 pairs of scans were included (Four were excluded following complete metabolic response, one patient had a non-FDG avid tumour, one had no post-CRT imaging, one had diffuse FDG uptake that could not be separated from normal tissues and one had an elevated blood glucose). The Pre40% and 50% of SUVmax volumes covered a mean of 50.8% and 30.3% of the GTV respectively. The mean% overlap of the 90%, 80%, 70%, 60% of SUVmax post-CRT with the Pre40% and Pre50% volumes were 83.3%, 84.0%, 83.7%, 77.9% and 77.8%, 69.9%, 74.5%, 64.8% respectively.

**Conclusions:**

Regions of residual metabolic activity following CRT can be predicted from the baseline FDG-PET and could aid definition of a biological target volume for non-uniform dose prescriptions.

## Background

At diagnosis, 30% of pancreatic cancers are locally-advanced and therefore unresectable despite the absence of metastatic disease [1]. Locally-advanced pancreatic cancer (LAPC) has a poor prognosis with median survival ranging from 5 to 11 months [2]. It can be treated by chemotherapy alone, chemoradiotherapy (CRT), or induction chemotherapy followed by CRT. Median overall survival (OS) is in the order of 11 months when CRT is used [3]. Recently, a large Phase III trial comparing induction chemotherapy prior to CRT and chemotherapy alone was closed at its first interim analysis as the addition of CRT did not offer a survival advantage (LAP07) [4]. Local progression at the original site of the tumour is the initial site of treatment failure in 25-29% of patients with LAPC [5,6]. A number of recommendations have been made for improving outcomes in LAPC – these include increasing the dose of radiotherapy to the whole, or part of the pancreatic tumour. Delivery of an adequate dose for local control to the pancreas is limited by the radiosensitivity of neighbouring organs - most notably the duodenum [7]. Selection of a biologically derived subvolume to boost within the tumour may make dose escalation possible without increasing normal tissue toxicity.

The best method of identifying regions within the pancreatic tumour that are more radioresistent is not known. A number of tracers that can be used in positron emission tomography (PET) which give information about tumour heterogeneity based upon cellular uptake and metabolism are available. The most widely used is ^18^ F-fluorodeoxyglucose (FDG). FDG uptake may give information about tumour biology. In a preclinical model, FDG avid tumours required an increase in radiation dose to improved local control rates, while tumours with low FDG-avidity did not benefit from an increased radiation dose [8], suggesting that FDG-PET may be an appropriate means of defining an area that would benefit from dose boosting.

We hypothesised that regions of high FDG uptake within the pancreatic tumour prior to CRT may identify areas of residual FDG avidity post-CRT. This has been suggested to be the case in non-small cell lung cancer (NSCLC) [9] and rectal cancer [10]. If residual FDG avidity six weeks after CRT for LAPC was spatially related to high FDG uptake on a baseline FDG-PET scan, this area could be thought of as ‘radioresistant’ and may serve as a means of delineating a biological target volume (BTV) that could be dose escalated.

## Methods

### Patient selection

Imaging from the first 17 patients enrolled into an ethically approved Phase II clinical study looking at the addition of the Akt-inhibitor nelfinavir to CRT (EUDRACT No: 2008-006302-42) was analysed. Inclusion criteria for the trial limited participation to those with locally advanced pancreatic adenocarcinoma or patients with resectable disease who were inoperable due to comorbidity with no evidence of metastatic disease.

### Chemoradiotherapy schedule

The treatment schedule has been described previously [11], in short, gemcitabine (300 mg/m^2^) and cisplatin 30 mg/m^2^ were administered on the Tuesday of the 1st, 2nd, 4th and 5th week of radiotherapy. In addition, nelfinavir was administered at a dose of 1250 mg twice daily from 3 days before until the last day of CRT. For each patient in this analysis, a 3D-conformal plan was made with a dose of 50.4 Gy in 28 fractions being delivered to the primary pancreatic tumour and elective regional lymph nodes with a sequential boost of 9 Gy in 5 fractions to the gross tumour volume (GTV) with a margin of 1 cm cranially, 2 cm caudally and 1.5 cm radially. A minimum of four 6–15 MV photon beams were used.

### Planning CT

Following fasting for 2 hours, the patient received 50 ml of water orally just prior to a contrasted-enhanced exhale breathold CT (CECT) followed by a 4DCT were performed on all patients to allow for radiotherapy planning. Patients were supine on a flat couch with knee rests, with arms above the head with a head support. The patient was scanned from above the dome of the diaphragm to the bottom of L4. A CT slice thickness of 2.5 mm was used.

### FDG-PET/CT scanning

FDG-PET/CT was performed at baseline and six weeks after completing CRT. All scans were performed on a GE Discovery 690 (GE healthcare, Buckinghamshire, UK). After fasting for 6 hours and ensuring that the blood glucose was <10 mmol/L, FDG was injected at a dose of 4 MBq/kg (up to 600 MBq). PET acquisition was started after an uptake time of 90 minutes. Patients were scanned immobilised in the radiotherapy treatment position to aid accurate image co-registration. The whole body from below the eyes to the mid-femurs were scanned. Scans were performed in 3D with a scan time of 4 minutes at each bed position. For the CT phase, 120 kV automA (max 250 mA), noise index 25.0 0.5 s/rotation, pitch 0.984:1, 3.75 mm slice width was used. Attenuation corrected PET images were used in the analysis.

### Planning software

The planning CT was loaded into the Eclipse software (version 10.0.42, Varian medical systems, Palo Alto USA). The gross tumour volume (GTV), as delineated by a radiation oncologist, was delineated on the exhale breath-hold CECT. The FDG-PET images were not registered with the planning CECT during GTV delineation. All GTVs were reviewed and approved by a second radiation oncologist to reduce inter-observer variability.

The CECT and FDG-PET images were imported into Mirada (Mirada Medical, Build 1.0.1.4, Oxford, UK) for non-rigid registration and then back into Eclipse for overlap fraction calculation.

### Thresholding based on standardised uptake values

Standardised uptake values (SUV) were calculated using an elliptical region of interest (ROI) drawn around the area of increased uptake within the pancreas that excluded the liver and kidneys. The maximum SUV (SUV_max_) within this ROI was recorded. Tumour segmentation was performed as a percentage of the SUV_max_ within the ROI. On the baseline FDG-PET this was performed at 40% and 50% of the SUV_max_. On the post-treatment images 60%, 70%, 80% and 90% of the SUV_max_ were used. A similar approach has been reported in non-small cell lung cancer [12], although the volumes produced by a % of SUVmax >50% on the pre-treatment FDG-PET were not considered for spatial correlation analysis as they generated very small regions of interest.

### Image co-registration

The Discovery 690 PET and CT images share the same intrinsic frame of reference, so the CT component was used for registration with the planning CT scan. In short, an automatic rigid registration, followed by a non-rigid registration, was performed within the Mirada software. Bony anatomy was visually checked to ensure that vertebrae were not misaligned and the quality of the entire registration was verified for gross errors. The deformation field from the verified CT-CT registration was then applied to the PET data and contours were propagated from the PET to the planning CECT.

### Assessment of spatial relationship of the SUV-derived subvolumes

The percentage of intersection between the volumes derived from the baseline 40% and 50% of SUV_max_ (Pre40% and Pre50% respectively) with the clinician defined GTV were assessed by quantifying the overlap between the two volumes and expressing that as a percentage of the GTV. The% intersection of the post-CRT volumes with the pre-CRT volumes was calculated and expressed as a % of the post-CRT volume. This method of defining an overlap fraction has been previously reported [12].

### Statistical analysis

Statistical analysis of the SUV_max_ pre- and post-CRT was performed using a two-tailed paired *t*-test within GraphPad Prism 5 (version 5.03, La Jolia, USA).

## Results

### Patient details

Of the 17 patients included, 4 had a complete metabolic response (cMR) at 6 weeks following CRT, one patient’s tumour was not FDG avid, one patient did not have a post-CRT FDG-PET, one patient had diffuse FDG uptake that could not be differentiated from surrounding normal tissue, one patient progressed with new liver metastases on the post-CRT FDG-PET and one patient had a serum glucose >20 mmol/L at both scanning time points, making image interpretation impossible. This left 9 pairs of scans that could be included in the analysis of the spatial distribution of pre- and post-CRT FDG uptake. Sixteen patients received CRT as planned. One patient’s treatment was discontinued after he had received 45 Gy to the GTV and elective lymph nodes because of a complication unrelated to this treatment.

### Assessment of treatment response

**Figure 1:**
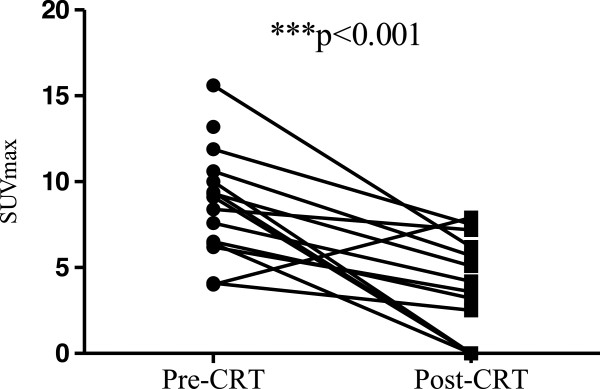
**Pre- and post-chemoradiotherapy (CRT) SUV**_**max **_**values.**

Pre and post-treatment SUV_max_ values for all patients are shown in Table 1. There was a significant decrease in SUV_max_ following CRT (pre-CRT median SUV_max_ 8, range 0–15.6, post-CRT median 3.6, range 0–7.9; p = 0.009, Figure 1).

**Table 1:** **Pre- and Post-treatment SUV**_**max **_**values and absolute volumes of the gross tumour volume (GTV) and volumes derived from segmentation of the pre- and post-treatment FDG-PET/CTs**

**Patient number**	**Pre-treatment SUV**_**max**_	**Post-treatment SUV**_**max**_	**Absolute volumes (cm**^**3**^**)**						
			**GTV**	**Pre40%**	**Pre50%**	**Post90%**	**Post80%**	**Post70%**	**Post60%**
1	7.6	4.2	57.6	28.9	19.8	0.2	1.6	6.9	14.7
2	8.4	7.2	80.2	31.6	18.7	0.1	0.3	1.7	16.7
3	6.4	mCR	14.5	7.4	3.8				
4	10.6	5.7	41.9	15.6	9.0	0.1	0.6	2.3	4.9
5	15.6	6.2	44.9	15.7	10.5	0.1	0.1	0.9	2.6
6	13.2	±	55.2	8.1	3.4				
7	6.2	3.6	38.0	22.4	12.1	0.1	0.3	1.6	4.4
8	11.9	7.5	27.2	13.4	5.4	0.1	0.6	1.4	2.6
9	4.0	7.9	28.6	§	§	§	§	§	§
10	9.1	mCR	49.9	34.2	9.2				
11	10.0	mCR	19.3	4.8	2.5				
12	9.4	mCR	14.0	6	3.2				
13	¢	¢	10.6	¢	¢	¢	¢	¢	¢
14	4.1	2.5	22.3	11.0	5.9	0.1	0.3	2.2	4.0
15	¤	¤	40.8	¤	¤	¤	¤	¤	¤
16	9.3	5.1	32.9	21.8	15.7	0.2	1.3	4	7.6
17	6.5	3.2	26.6	19.0	11.8	0.2	1.6	5.5	11.4
Median	8.0***	3.6*** P < 0.001							
Mean^			41.3	19.9	12.1	0.1	0.7	2.9	7.7

mCR metabolic complete response. ¢ Not FDG avid. § Diffuse FDG uptake making delineation of a ROI impossible. ¤ Elevated serum glucose, scans therefore could not be interpreted. ± Not done. *Difference in pre- and post-treatment SUV_max_ p = 0.0006. ^only values included in spatial correlation analysis included.

### Relationship of the pre-CRT FDG-PET derived subvolumes and the clinician defined GTV

**Figure 2:**
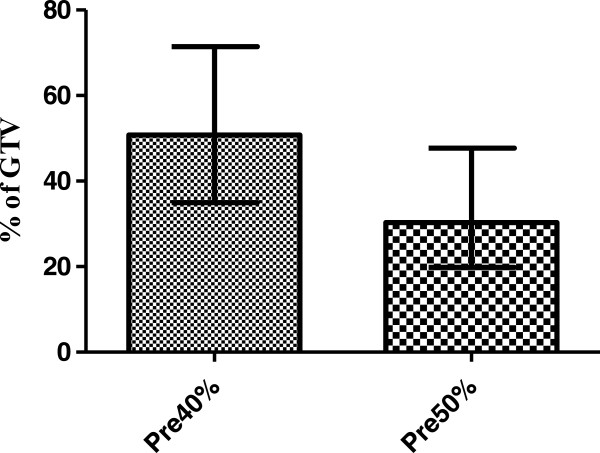
**Pre-treatment volumes defined by 40% of SUV**_**max **_**(Pre40%) and 50% of SUV**_**max **_**(Pre50%) expressed as proportion of the GTV (mean and range).**

The mean GTV volume was 41.3 cm^3^ (range 22.3-80.2 cm^3^), mean Pre40% and Pre50% volumes were 19.9 cm^3^ and 12.1 cm^3^ respectively. The intersection between the Pre40% volume the GTV was a mean of 50.8% (range 35.0-59.0%) of the GTV, while the Pre50% volume was a mean of 30.3% (range 19.9-34.4%) of the GTV (Figure 2).

### Degree of agreement between the post-CRT areas of FDG uptake and the pre-CRT SUV40% and SUV50%

**Figure 3:**
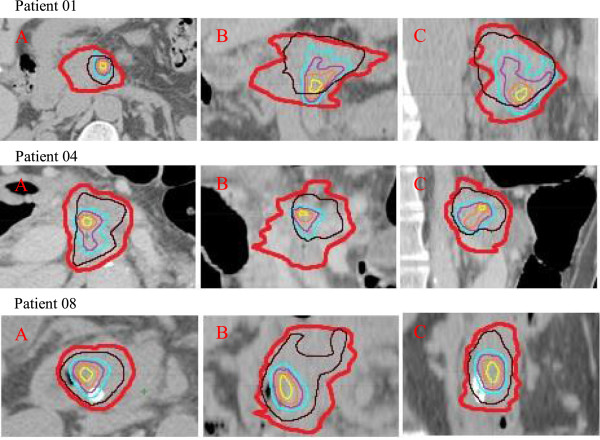
**Relation of the GTV (red) and Pre40% (brown) to the Post60% (cyan), Post70% (purple), Post80% (orange), Post90% (yellow) in three representative patients.** (**A**. axial **B**. coronal **C**. sagittal images).

**Figure 4:**
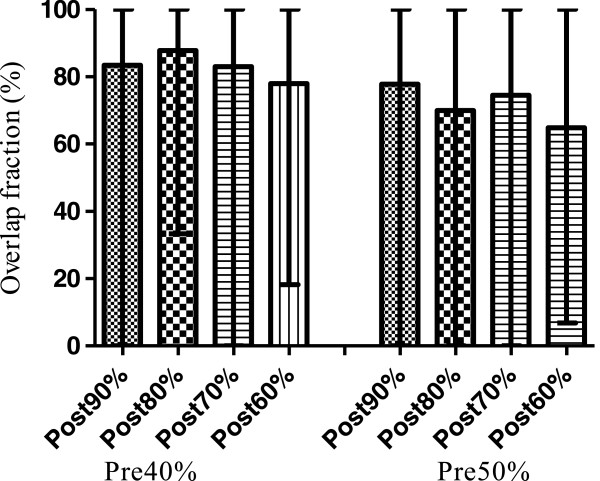
**Degree of overlap between the Pre40% and Pre50% subvolumes and the post-CRT subvolumes.** Bars represent mean% intersection of the post-treatment volume with the pre-treatment volume. Error bars represent the range.

Figure 3 demonstrates how the volumes derived from the pre-CRT FDG-PET relate to the post-CRT volumes and to the GTV on the planning CECT. Patient 1 demonstrates the effect of tumour shrinkage on the relationship between the pre and post-CRT volumes. While most of the residual metabolic activity is contained in the Pre40% volume, the tumour moved in a caudal direction following CRT. Patient 4 shows an excellent correlation between pre- and post-CRT metabolic activity with all FDG avid areas being contained within the Pre40% volume and the GTV. Patient 8 also shows an excellent agreement between the pre- and post-CRT metabolic activity, but the Pre40% volume covers a large proportion of the GTV (49.3%). Figure 4 demonstrates the % of the residual FDG uptake volumes contained within the Pre40% and Pre50% volumes averaged over all the patients. The absolute values can be found in Table 2.

**Table 2:** **Degree of overlap between the post-treatment FDG-PET derived volumes and the pre-treatment 40% and 50% of SUV**_**max **_**volumes**

	**% intersection of the following volumes**							
	**Pre-treatment 40% of SUV**_**max**_				**Pre-treatment 50% of SUV**_**max**_			
Patient	Post90%	Post80%	Post70%	Post60%	Post90%	Post80%	Post70%	Post60%
1	100.0	87.5	87.0	83.0	50.0	68.8	66.7	64.0
2	100.0	100.0	100.0	68.9	100.0	100.0	100.0	54.5
4	100.0	100.0	100.0	95.9	100.0	16.7	87.0	81.6
5	100.0	100.0	100.0	100.0	100.0	100.0	100.0	88.5
7	0.0	0.0	6.3	18.2	0.0	0.0	0.0	6.8
8	100.0	100.0	100.0	100.0	100.0	100.0	100.0	100.0
14	100.0	100.0	100.0	90.0	100.0	100.0	81.8	70.0
16	100.0	100.0	100.0	93.4	100.0	100.0	95.0	82.9
17	50.0	68.8	60.0	51.8	50.0	43.8	40.0	35.1
Mean	83.3	84.0	83.7	77.9	77.8	69.9	74.5	64.8

## Discussion

Delivering an explicitly non-uniform radiotherapy dose distribution, such as boosting a biological subvolume, to improve rates of local tumour control is the subject of much interest. Treatment outcomes in LAPC are poor and approximately 1 in 4 patients have the initial site of treatment failure within the GTV [5,6]. The results of this study suggest that areas of residual metabolic activity following CRT can be predicted by looking at volumes of ≥40% of SUV_max_ on the pre-CRT FDG-PET scan. This supports the hypothesis that areas of increased FDG uptake prior to treatment have a degree of chemo- and radioresistance that had been suggested from preclinical [8] and rectal cancer data [10]. Models of tumour heterogeneity suggest that the optimal dose prescription can be approximated using only a few dose levels – if the compartment boundaries and prescribed dose levels are well chosen [13].

The Pre40% volume seems to be the most pragmatic to use for biological target volume (BTV) definition. It covers most of the residual FDG-avid areas and only covers an average of 50.8% of the GTV, so dose escalation without increasing toxicity to normal tissues may be feasible. The Pre50% volume has been recommended as a suitable boost volume in NSCLC as the Pre40% volume covered almost all of the tumour [12]; the authors proposed that, a mean overlap fraction between the Pre50% and the Post90% volume of 70.4% supported the use of the Pre50% volume for BTV definition. In our study, the mean overlap fraction of the Pre40% volume with the Post90% volume was 83.3%, with 100% of the volume being included in all but 2 patients while covering an average of only 50.3% of the GTV.

Absolute SUV values for volume delineation (data not shown) were also applied on the pre- and post-CRT FDG-PET images. Using an SUV value of 2.5, it was difficult to differentiate from the metabolic activity in adjacent normal tissues – most noticeably in the liver and small bowel. It was therefore decided that values relative to the SUV_max_ should be used to segment the tumours. A number of thresholds have been used to segment tumours of various types to aid target volume definition based on FDG-PET SUV_max_. The percentages most commonly used are 40% and 50% of the SUV_max_ (reviewed in Biehl et al. [14]). These values were therefore used in this study. Another study used values from 34%-70% of the SUV_max_ on the pre-treatment FDG-PET to segment the tumour [9]. We found that values lower than 40% of the SUV_max_ produced large volumes that extended beyond the region of interest and that using a threshold ≥60% of SUV_max_ produced a volume that did not sufficiently overlap with areas of residual metabolic activity. Post-CRT thresholding was done at levels ≥60%. As the SUV_max_ had decreased during CRT segmentation using a threshold <60% of SUV_max_ led to the inclusion of normal tissue in the volume. The smaller uptake volume on the post-CRT FDG-PET will make the SUV appear reduced due to the recovery coefficient of the scanner. This may also explain why higher relative thresholds were required to produce volumes that were contained by the residual tumour mass on the post-CRT imaging.

The commercially available non-rigid registration algorithm employed here has been validated for a number of clinical tumour sites [15-17] and been shown to give residual registration errors typically less than 3 mm, depending on the clinical site and the magnitude of initial displacement. All registrations were checked visually by a second expert radiation oncologist.

The change in both the size and location of the target further highlights the need for adaptive planning during radiotherapy for LAPC – particularly if a dose boost to a metabolically active subvolume is to be considered. Further investigation into how the tumour shrinks throughout treatment, perhaps with functional imaging with adaptive thresholding, may increase confidence in the location of the BTV within the GTV.

Of the 17 patients who had paired FDG-PET scans, only 9 pairs of scans were included in this analysis. Four patients had a metabolic complete response (mCR), so increasing the dose to the FDG-avid area would not have improved the imaging outcome. Care should be taken to avoid regarding mCR as ‘cure’. Residual FDG uptake was taken as an end-point in this study as it was thought to equate to a radioresistant subvolume within the tumour. The aim of any further dose escalation should not end at the establishment of mCR, but rather pathological complete response and long-term tumour control. While a fall in SUV_max_ greater than the median value in a cohort of patients was found to be predictive of an improved overall and progression free survival [18], no large-scale trials have definitively demonstrated that a mCR equates to an overall survival advantage in LAPC. There are no published data to suggest that patients who achieve mCR will not derive benefit from an increased dose. Four patients in this study did achieve mCR and could potentially not derive any benefit from dose escalation. This reinforces our belief that any future dose escalation studies should be isotoxic and that further evaluation of the impact metabolic response has on the prognostic utility of FDG-PET in LAPC is required.

The best method of risk stratifying which patients would most benefit from an intensification of therapy, accepting the inherent increase in treatment associated morbidity, remains unclear. Patients with intact Smad4 (Dpc4) expression tend to have a local pattern of progression compared to those with Smad4 (Dpc4) loss who tend to have a distant pattern of progression [5]. This suggests that tumours with intact Smad4 may benefit from an increased radiotherapy dose. While we have demonstrated that areas of residual FDG avidity can be identified from pre-treatment imaging, FDG-PET alone may not be the most robust way of risk stratifying patients. Other imaging modalities, for example ^18^ F-fluormisonidazole-PET/CT, ^18^ F-fluorthymidine-PET/CT, and diffusion weighted MRI, may also have a role in defining a radioresistant target for dose escalation and merit further investigation. It is likely that a combination of imaging and biological markers will offer the greatest discriminatory value.

While an increase in radiotherapy dose has been shown to impact upon survival [19], this is at the expense of increased treatment associated toxicity [20,21]. Targeting a subvolume within the GTV may allow an increase in dose without compromising normal tissue constraints, particularly if 4D imaging was used to allow for a personalised, reduced margin.

## Conclusion

Thresholding a pancreatic tumour using 40% of the SUV_max_ on baseline FDG-PET/CT identifies areas of residual metabolic activity seen on a post-CRT FDG-PET/CT. While the volume derived from 40% of the SUV_max_ predicts a priori the geographical location of residual metabolically active tumour post-CRT in most patients, this analysis was only possible in approximately 50% of the patients. Any future dose escalation studies that use this subvolume should be isotoxic to avoid increasing treatment associated toxicity in those patients who may not derive therapeutic benefit.

## Consent

Written informed consent was obtained from all patients for the publication of this report and any accompanying images.

## Competing interest

James M. Wilson is funded by CR-UK & EPSRC Cancer Imaging Centre in Oxford, in association with the MRC and Department of Health (England). Maria A. Hawkins is funded by the MRC (Medical Research Council).

## Authors’ contribution

JMW carried out the image registrations, target volume definitions, PET image thresholding including overlap fractions and drafted the manuscript. SM delineated target volumes and is the principal investigator of the clinical trial. KYC assisted in patient positioning and data management. TB delineated target volumes and designed the clinical trial. MP supervised the work and revised the final manuscript. MH supervised the work, reviewed the target volumes and image coregistration and revised the final manuscript. All authors read and approved the final manuscript.
